# A sequential model for the structure of health care utilization

**DOI:** 10.1371/journal.pone.0176657

**Published:** 2017-05-12

**Authors:** Wolfram J. Herrmann, Alexander Haarmann, Anders Bærheim

**Affiliations:** 1 Institute of General Practice, Charité-Universitätsmedizin Berlin, Berlin, Germany; 2 Institute of General Practice and Family Medicine, Otto-von-Guericke-University of Magdeburg, Magdeburg, Germany; 3 Department of Global Public Health and Primary Care, University of Bergen, Bergen, Norway; The Chinese University of Hong Kong, HONG KONG

## Abstract

Traditional measurement models of health care utilization are not able to represent the complex structure of health care utilization. In this qualitative study, we, therefore, developed a new model to represent the health care utilization structure. In Norway and Germany, we conducted episodic interviews, participant observation and a concurrent context analysis. Data was analyzed by thematic coding in the framework of grounded theory. Consultations do very often not only have one single reason for encounter. They are usually not independent events but form part of consultation sequences. We could find structural differences between Norway and Germany regarding the flow of information between consultations and which providers are involved in health care in what way. This leads to a sequential model, in which health care utilization is seen as sequences of consultations. Such health care utilization sequences consist of nodes which are connected by edges. Nodes represent patient-provider contacts and edges depict the flow of information. Time and the level of health care providers are dimensions in the model. These sequences can be described by different measures and aggregated on population level. Thus, the sequential model can be further used in analyzing health care utilization quantitatively, e.g., by using routine data.

## Introduction

Health care utilization is an important aspect to describe and understand health care systems. Often it is researched in the context of inequality of access and utilization regarding differences in socio-economic status, education, gender, race, specific illnesses, or regions [1–5]. It also plays an important role regarding inappropriate utilization, e.g. in the German or Spanish health care system [6, 7]. Health care utilization and its determinants are also important for health care planning [8, 9].

Health care utilization differs spatially, between countries as well as inside countries [5, 10, 11]. It can for instance be shown that health care utilization rates in Norway are more than three times smaller than in Germany [12]. However, neither morbidity patterns and life expectancy nor the relative number of physicians differ between these two countries; some differences can be found with regard to the relative number of family physicians and the health care expenditure (cf. Table 1) [13].

**Table 1:** Number of physicians and health care expenditure compared between Germany and Norway [13].

	Germany	Norway
Physicians per 100,000 inhabitants	382.4	371.8
Family physicians per 100,000 inhabitants	65.8	90.2
Health care expenditure of GDP	11.3%	9.0%

Health care utilization can be measured regarding health care services in general [5] or of defined distinctive services [4, 14]. There are three different approaches for measuring health care utilization which are commonly used: if a service was used at all, how often a service was used per time interval and the overall cost of health care services. In a review of studies using Andersens’ behavioral model [15] Babitsch et al. reported that the majority of studies used binary outcomes for health services research, thus, if a service has been used or not [16]. This approach has also been used, e.g., in the measurement studies of Jordan et al. and Reijneveld [17, 18]. This approach is especially adequate for questions of access to health care. A second approach are the numbers of use of services per time interval, e.g. number of emergency room visits, number of hospitalizations, number of hospital nights, number of physician visits most often per month or per year. This approach of rates has been used in several measurement studies [19–23]. A third approach combining services is to measure the overall costs [5, 21]. Models for measuring health care utilization taking more complex structures of health care utilization into account are not commonly used.

These measurements of health care utilization are then applied to causal models, such as Andersens’ behavioral model. In Andersen’s behavioral model, which exists in many different versions, factors such as need and enabling factors are associated with health care utilization measures [15]. A different approach in health care utilization research is to look at individual decision processes how to decide on utilizing health care having a particular health care problem [24]. In this approach health care utilization is modeled as single (usually independent) events.

Starting from the health care utilization differences between Germany and Norway, our goal was to develop exploratively a new measurement model for health care utilization which might be better capable to represent complex health care utilization differences.

## Method

We chose a qualitative study design. As cases we opted for Norway and Germany, countries with similar morbidity and mortality patterns. Furthermore, health care is provided mainly by self-employed family physicians in both countries. The German health care system is a Bismarckian system with mandatory health care insurance for about 90% of the population. In ambulatory care, family physicians and specialists are working self-employed and usually in small scale practices. There are no co-payments for consultations but for medication and there is free access to all ambulatory physicians [25]. The Norwegian health care system is a state driven system financed by taxes and co-payments. Mainly self-employed family physicians practice in group practices. Every patient is registered with a family physician. Specialists are mainly working in outpatient clinics at hospitals. There is gatekeeping in place, which means that patients have to be referred by a family physician to a specialist [26].

To analyze patients’ concepts and behavior in the interplay with the health care system, we chose a threefold methodological approach: First, we conducted episodic interviews with patients, a type of qualitative interview, which consists of narrative and abstract questions. Second, we conducted participant observations in primary care practices, mainly following consultations in the role of a guest. Third, we compared emerging factors between the two health care systems and checked for existing evidence of their effect.

Access to the field was granted via eight primary care practices, four in Germany and four in Norway. In both countries, two of them were situated in rural and two in urban settings. They were diverse regarding their size. In each of these practices we conducted several blocks of participant observation over the course of one week. Overall, we observed 400 consultations.

In each of the practices we recruited potential interviewees by a short questionnaire handed out to all patients on two or three days. The questionnaire contained questions on age, gender, number of chronic conditions and number of visits to the GP. Out of this pool of potential interviewees we chose the Norwegian interviewees by theoretical sampling and matched the German interviewees accordingly regarding the four categories of the questionnaire and if the visited practice was a rural or urban one. Overall, we interviewed 40 patients. An overview of the socio-demographic characteristics of the sample can be found in Table 2.

**Table 2:** Socio-demographic information of our sample of interviewees.

	Germany		Norway	
	urban	rural	urban	rural
female	5	4	4	5
male	7	4	5	6
range of years of birth	1935–1990		1931–1987	
average year of birth	1957		1957	
Σ	20		20	

Data was analyzed by thematic coding in the framework of grounded theory [27, 28]. First, selected interviews and observation protocols were coded line by line by several researchers [29]. Then we developed a thematic structure for the codes in joint workshops. A thematic structure is a hierarchical ordering of codes into categories. Consecutively, all interviews were coded and memos written by the help of the online-tool Dedoose. During the process of coding, the thematic structure was further refined. Four main findings regarding the structure of health care utilization emerged during the process of analysis. For all these findings we looked for confirming and contradicting data in the overall material.

The study has been conducted according to the principles expressed in the Declaration of Helsinki. All interviewees and observed patients gave written informed consent. The study has been approved by the ethical review committee of the medical faculty of the Otto-von-Guericke-University of Magdeburg and by the Regional Committee for Medical and Health Research Ethics in Western Norway (REK Vest). A more detailed description of the methodology is published in the study protocol [30].

## Results

### Consultations are often not due to a single reason

During participant observation in primary care practices in Norway and Germany, we could observe that consultations were frequently not owed to an acute single reason for encounter. Most often, especially in patients with chronic conditions, there were several “collected” reasons for encounter or no explicit reason at all. This is even more illustrated by the common behavior of Norwegian patients to bring a list of topics they want to talk about with the doctor.

This can be found back in the interviews as well. The following German participant, for instance, is talking about seeing his doctor regularly and collecting topics he wants to raise:

*I had to go to the doctor, because, all the time, I’ve been taking blood pressure medication. Thus, he wanted to see me, asked me to come in intervals. Maybe about once a month. And I liked that, liked that very much. And if you had something else, you could tell him during that occasion as well. He has seen me about every quarter of a year. He has given me a new appointment every time*.

(D14)

An important aspect of these follow-up visits in both countries is monitoring, as is illustrated by this quote of a Norwegian patient:

*I visit him [(the family physician)] and get these measurements done. Every now and then he measures my blood pressure, every now and then he takes blood samples and sends them to the university hospital. And so on. And usually we agree on that I will return to see him in about four, five weeks. Thus, I make an appointment with the medical assistant before I go*.

(N08)

Such follow-up visits are often induced by the doctor, who wants to monitor the patients’ health status:

*And I have to say, we have such a rhythm: She wants to see me every four weeks, no matter if I have any complaints or not. Hence, she wants to be up to date and to see me every few weeks. And I must admit, otherwise, I wouldn’t visit the doctor*.

(D16)

Thus, consultations do very often not have just one single reason for encounter, while they at the same time often originate in the physician’s desire to monitor the health status of the patient.

### Consultations are events in sequences of consultations

In line with this observation, we found during our participant observation that most consultations cannot be understood in themselves: Just from following the consultation from its start to its end, it is with few exceptions not comprehensible on its own, because it builds on what has previously happened and is known to GP and patient. This observation is strengthened by the common behavior of family physicians to explain the background of the patient’s story and what had happened in previous consultations to the observer before or after the consultation. This highlights that the consultation itself is usually just one single event in a longer process, thus it is not an independent event.

Additionally, a consultation is not only linked to those consultations and events which preceded this consultation, but very often a follow-up consultations is already appointed at the end of the consultation. Thus, a consultation is most often an event in a sequence of consultations. This can be highlighted by the following quote of a patient who is summoned by his GP quarterly:

*Why do I have to go so often to the doctor? Because he summons me, I do not go there on my own will. […] Why does the GP want to see me every quarter of a year in his surger? I can understand that I shall go annualy to the urologist because of preventive services*.

(D14)

Thus, consultations are usually neither due to a single reason nor closed events, but events in a sequence of consultations.

### Structural differences in referrals and flow of information

Regarding how these consultations are connected, we could observe structural differences between Norway and Germany concerning referrals and the flow of information.

In the Norwegian health care system, referrals by the family physician are obligatory:

*However, that’s then the Norwegian system. That’s how it is in Norway, if you want to or not. That’s how it is for every one, you don’t have the right to go directly to a specialist. You must go via a family physician anyhow*.

(N04)

In Norway, referrals are (electronic) letters sent from the family physician to a designated specialist. These letters contain detailed information, e.g. about the urgency of the problem. Depending on this information the specialist decides in what order she sees the patients and sends everyone on the list the date of their appointment.

In the German health care system, referrals are facultative:

*I’m happy that it’s over with the rule that you had to go to the family physician all the time first. Now I am able to decide on my own if I go to a doctor who says, no that’s nothing [serious] or if I go to a specialist who has different possibilities to look closer and find maybe something*.

(D10)

In Germany, referrals are short forms which are handed out to the patient. They contain usually very sparse if any information. The patient has then to organize an appointment with a specialist.

While information from specialists back to family physicians flows mainly by electronic letters in Norway, this information is paper-based and sent by post or via the patient in Germany. However, often this information is missing, which makes the integration of findings by different physicians difficult.

Thus, the flow of information between the different consultations differs between Norway and Germany.

### Differences in which specialist providers are involved

While in Norway a gatekeeping system is in place and other providers than GPs are sparsely in ambulatory care, specialists in ambulatory health care is widespread in Germany:

*The last time I visited a doctor was the dentist, and for check-up […] the second last time, maybe more interesting was at the oncologist for aftercare, because I have cancer. […] and before that I visited the urologist; he checked something because of the surgery and cancer […], and before that I visited the angiologist […] who ran tests. And before that I visited the cardiologist, who did tests as well*.

(D14)

Especially, for patients with chronic conditions health care is shifted towards specialists, which is also highlighted by this quote of a patient with renal failure:

*That’s why I have nothing to do with the family physician, nothing. I receive medication and those things just from the nephrologist. As far as I’m concerned, yeah. And otherwise, the family physician—almost nothing*.

(D12)

This can be contrasted to findings from participant observation in Norway: A family physician told she has to care even for highly disabled chronically ill children, and she only gets advice, e.g. by phone, from the pediatrician.

Thus, not only the connection between the consultations but also the level and kind of provider these consultations differ between Norway and Germany.

### A new derived model for describing the structure of health care utilization

Hence, we can conclude that a new model representing the structure of health care utilization has to include consultations as events in sequences of consultations, the dimension of time, the different providers involved in health care, and the different flows of information between the providers. Thus, the components of the model we want to propose are:

- health care utilization as a sequence of consultations
- nodes represent patient-provider contacts
- edges connect these nodes and depict the flow of information as
  – referral
  – medical report
  – follow-up appointment
- time as dimension
- level of health care provider as dimension

**Fig 1:**
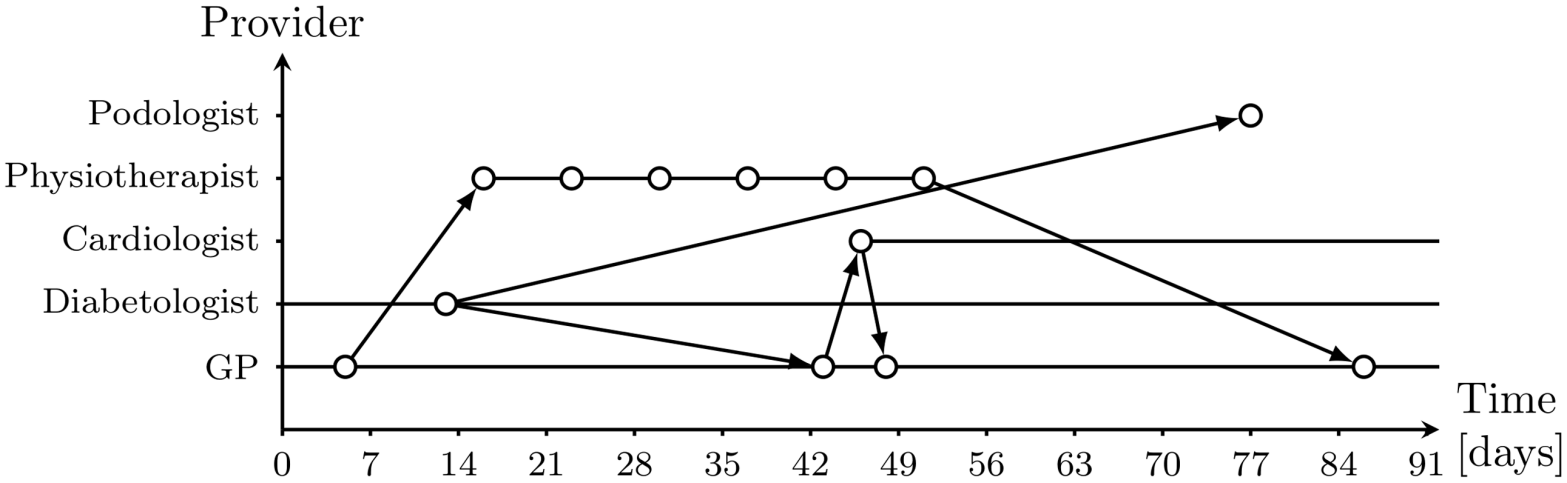
A fictitious example of a health care utilization sequence.

Hence, health care utilization is first of all seen as individual health care utilization sequences which can be summarized by measures and aggregated on population level. Fig 1 gives a fictitious example of such an individual health care utilization sequence. As one can see, these sequences consist of superimposed patient pathways.

Based on the qualitative findings, e.g., the differences in flow of information between the Norwegian and Germany health care system, we propose as summary measures for individual health care utilization sequences: The qualitative result hint at differences in the length of the interval between visits, both, for GPs and other specialists. Thus, the length of the interval between visits is a summary measure. As described above, also the number of health providers involved varies, which is another summary measure. Another difference between the health care in Norway and Germany is the flow of information. Hence, another summary measure is the continuity of the flow of information. The continuity of the flow of information describes how many patient-provider-contacts are connected by flow of information to preceding patient-provider-contacts. For some patients, there are threads of health care provision which are only loosely connected with each other: E.g., a patient may visit regularly his orthopedist without a prior referral and is referred by him to physiotherapy; this patient also visits regularly his family physician, but there is only low nor no interaction between his family physician and his orthopedist.

## Discussion

Our qualitative findings highlight the importance to look at health care utilization not as a count of contacts but as events on patient pathways. We call these pathways health care utilization sequences. In the model we propose, the nodes of these sequences are patient-provider-contacts connected by the flow of information as edges.

Because it is a qualitative study we did not aim for representativity of our findings but for generalizability of the found structures. Thus, the quantitative applicability and distribution has to be evaluated in follow-up studies. The found differences are closely related to underlying aspects of the respective health care systems. However, we deem it important to highlight which aspects of the health care system contributes to which specific structures of health care utilization and in what way instead of drawing any unspecific relationship to the overall system. The derived model shall enable further research on such connections.

Our findings that consultations are often not just owed to one specific reason for encounter are in line with the Norwegian recommendation for family physicians to ask for all reasons in the opening phase of a consultation [31]. The habit of frequent follow-ups of patients with chronic conditions found in the German part of this study are in line with the findings of secondary data analysis of German patients, where patients with chronic conditions commonly had 25 or more contacts per year [10, 32, 33].

Our findings are derived from a study conducted in Norway and Germany, two countries with different health care systems; one is a state-driven system and one a Bismarckian. Both health care systems have in common to have mainly self-employed family physicians and both countries are industrialized affluent countries. However, the general model we derived can also be transferred to different types of health care systems, such as in the US, because it is a generic one starting from patients’ experience. The model we describe could be also extended to countries where other providers such as traditional healers are involved. They can be denoted as providers.

A limitation of our study is that only participants who at least visit the doctor once could be included into the study because of the recruitment by GP surgeries. However, also participants who visit doctors very rarely could be included. A strength of our study is that it consisted of different methodological approaches, episodic interviews and participant observation. The findings in both approaches were triangulated and, thus, have a higher validity.

This new measurement model can be directly related to the common models presented in the introduction. From a health care utilization sequence, both, counts of number of visits and a binary variable if a provider has been visited can be directly deduced. However, the sequence contains much more information of the course of health care over time.

The here introduced health care utilization sequences can be described mathematically as directed networks or graphs. This opens up the possibility to use algorithms from graph theory to analyze these sequences. For example, for the continuity of the flow of information, one needs to check for the connectivity of all nodes with the in time preceding nodes. This can be done by the according graph theory algorithms for connectivity.

## Conclusions

Our findings show that health care utilization goes beyond counts of consultations. Because consultations and individual events of utilization are not independent of each other, summative rates are often oversimplifying. The course of health care utilization is more complex, thus, we prefer to call it health care utilization structure. This health care utilization structure can be represented by health care utilization sequences. These sequences of individual patients can on the one hand establish a framework for further analyses of health care utilization patterns. On the other hand these sequences can be aggregated on population level, e.g., regarding inhabitants of a designated geographical area.

The sequential model should be applied in a quantitative research design in a next step. Especially routine date seem to be an appropriate source for extracting the necessary information to construct health care utilization sequences. On individual and aggregated level, it is then possible to look for determinants of either patterns of sequences or determinants of characteristics of the sequences. Additionally, these sequential approach is going to allow researching the influence of health care characteristics, such as the role of the GP, on outcomes, such as hospitalization rates. For analyzing and simulating such sequences the graph character of the sequences and computational advances in graph theory can be used.
